# Bone Morphogenetic Protein (BMP)4 But Not BMP2 Disrupts the Barrier Integrity of Retinal Pigment Epithelia and Induces Their Migration: A Potential Role in Neovascular Age-Related Macular Degeneration

**DOI:** 10.3390/jcm9072293

**Published:** 2020-07-19

**Authors:** Ahmed S. Ibrahim, Khaled Hussein, Fang Wang, Ming Wan, Nancy Saad, Maamon Essa, Ivana Kim, Akbar Shakoor, Leah A. Owen, Margaret M. DeAngelis, Mohamed Al-Shabrawey

**Affiliations:** 1 Department of Ophthalmology, Visual, and Anatomical Sciences, Department of Pharmacology, Wayne State University, Detroit, MI 48201, USA; 2 Department of Biochemistry, Faculty of Pharmacy, Mansoura University, Mansoura 35516, Egypt; 3 Department of Medicine and Surgery, Oral and Dental Research Division, National Research Centre, Cairo 12622, Egypt; hussein.k.nrc@gmail.com; 4 Department of Oral Biology and Diagnostic Sciences, Augusta University, Augusta, GA 30912, USA; tealiking@aliyun.com (F.W.); brightwan@sina.com (M.W.); Abdelhay@ualberta.ca (N.S.); maamonessa@gmail.com (M.E.); 5 Department of Traditional Chinese Medicine, School of Medicine, Jianghan University, Wuhan 430199, China;; 6 Dental school, University of Alberta Canada, Edmonton AB T6G 2R3, Canada; 7 Department of Medical Biochemistry, Mansoura Faculty of Medicine, Mansoura University, Mansoura 35516, Egypt; 8 Retina Service, Harvard Medical School, Massachusetts Eye and Ear, Boston, MA 02115, USA; ivana_kim@meei.harvard.edu; 9 Department of Ophthalmology and Visual Sciences, University of Utah, Salt Lake City, UT 84112, USA; akbar.shakoor@hsc.utah.edu (A.S.); Leah.Owen@hsc.utah.edu (L.A.O.); mmdeange@buffalo.edu (M.M.D.); 10 Department of Population Health Sciences, University of Utah School of Medicine; Salt Lake City, UT 84132, USA; 11 Department of Ophthalmology, Jacobs School of Medicine and Biomedical Engineering, University at Buffalo SUNY, and the VA Western New York Healthcare System, Buffalo, NY 14215, USA; 12 Department of Cellular Biology and Anatomy, Augusta University, GA 30912, USA; 13 Department of Ophthalmology and Culver Vision Discovery Institute, Augusta University, Augusta, GA 30912, USA; 14 Department of Anatomy, Mansoura Faculty of Medicine, Mansoura University-Egypt, Dakahlia Governorate 35516, Egypt

**Keywords:** age related macular degeneration (AMD), BMP4, BMP2, retinal pigment epithelial cells (RPE), ARPE-19

## Abstract

Disruption of retinal pigment epithelial (RPE) barrier integrity and RPE migration are hallmark features in neovascular age-related macular degeneration (nAMD), but the underlying causes and pathophysiology are not completely well-defined. Herein, we aimed to evaluate the effect of bone morphogenetic proteins (BMPs) on the barrier function and migration of RPE. In particular, we investigated the role of BMP2 and BMP4 in these processes as our analysis of RNA-sequencing (seq) data from human donor eyes demonstrated that they are highly differentially expressed BMP members in macular RPE/choroid versus macular retina. We used electrical cell-substrate impedance sensing (ECIS) system to monitor precisely in real time the barrier integrity and migration of ARPE-19 after treatment with various concentrations of BMP2 or BMP4. Immunofluorescence was also used to assess the changes in the expression and the organization of the key tight junction protein, zona occludens (ZO)-1, in ARPE-19 cells under BMP2 or BMP4 treatment. This was followed by measuring the activity of matrix metalloproteinases (MMPs). Finally, RNA-seq and ELISA were used to determine the local and circulating levels of BMP2 and BMP4 in retinas and serum samples from nAMD donors. Our ECIS results showed that BMP4 but not BMP2 decreased the transcellular electrical resistance (TER) of ARPE-19 and increased their migration in comparison with control (vehicle-treated cells). Furthermore, immunofluorescence showed a disorganization of ZO-1 in BMP4-treated ARPE-19 not in BMP2-treated cells or vehicle-treated controls. This effect of BMP4 was associated with significant increases in the activity of MMPs, specifically MMP2. Lastly, these results were corroborated by additional findings that circulating but not local BMP4 levels were significantly higher in nAMD donor samples compared to controls. Collectively, our results demonstrated unreported effects of BMP4 on inducing RPE dysfunction and suggest that BMP4 but not BMP2 may represent a potential therapeutic target in nAMD.

## 1. Introduction

Age-related macular degeneration (AMD) is a progressive neurodegenerative disease causing vision loss and even blindness in geriatric population over age of 60 [1]. AMD begins as a clinically undetectable disease with the thinning of macula, a light sensitive tissue in the center of the retina needed for seeing fine detail. As this early stage of the disease progresses, a noticeable accumulation of debris known as drusen becomes more visible upon ophthalmoscopic examination as yellow spots beneath the retina [2]. This early stage of the disease is known as dry AMD and patients at this stage are at high risk for developing both the end stages of disease, including geographic atrophy (GA), in which the retinal pigment epithelial cell (RPE) is compromised and contributes to the death of the overlying photoreceptors [2]. Patients may also progress to neovascular AMD (nAMD), which is characterized by the abnormal growth of new fragile blood vessels from the choroid into the subretinal space in a process called choroidal neovascularization (CNV), resulting in hemorrhage and blindness [3] (for comprehensive reviews on the clinical manifestations of AMD please see [4,5,6,7,8]).

It is expected that the worldwide number of people with AMD will reach 288 million by 2040 [9] which places an enormous burden on the health care system and society. Existing therapeutic strategies for AMD includes intraocular injections of anti-vascular endothelial growth factor (VEGF), such as ranibizumab (Lucentis), bevacizumab (Avastin), and aflibercept (Eylea), laser photocoagulation, photodynamic therapy (PDT), and an implantable telescope [10,11,12,13]. While many patients respond well to the anti-VEGF treatments for neovascular AMD, a subset of patients do not. These modalities may be limited by their invasiveness and burden to the patient and treating ophthalmologist. Additionally, there is yet no interventional therapeutic for disease progression to the late stages of AMD or cure [14]. Therefore, there is an urgent need, to find innovative mechanistic avenues to treat or prevent AMD.

The preservation of RPE function is a primary goal for AMD treatment [15]. This is due to the important functions that RPE cells carry out, including phagocytosis of outer segments of photoreceptors, metabolism of vitamin A, and VEGF secretion which maintains the health of choriocapillaris endothelium [5,16]. Most importantly, RPE cells control the passage of molecules between retina and systemic circulation together with Bruch’s membrane, constituting the outer blood retinal barrier (oBRB) [14]. With normal aging, RPE cells undergo substantial changes that affect their functionality in maintaining the integrity of oBRB as well as the homeostasis of photoreceptors and the choroid, triggering the development of AMD and its progression to the advanced stages [17,18,19]. It has been demonstrated that increased expression of VEGF by RPE cells and their intraretinal migrations occurred in various AMD stages, most commonly in human eyes with advanced AMD lesions [20,21,22]. Nevertheless, the triggering mechanisms of RPE dysfunction with aging are still incompletely understood.

In recent years, bone morphogenetic proteins (BMPs) have been identified in non-osseous tissues, such as endothelia and RPE cells, but their functions and signaling there are poorly understood [23,24]. Mathura et al. [25] have found BMP2 and BMP4 mRNA at a high level of expression in the adult RPE cells compared with other tissues. However, the exact role of BMP2 or BMP4 in regulating adult RPE functions appears to be contradictory; while it has been suggested that BMP-2 and BMP-4 might act as negative growth regulators for RPE [25], others have reported that BMP-4 may be involved in the ocular angiogenesis via stimulation of VEGF secretion by RPE cells [26]. Therefore, the aim of the current study was to evaluate the direct effects of BMP2 and BMP4 using a multi-pronged approach on the barrier integrity and migration of RPE cells and to measure their circulating and tissue levels in donor patients with neovascular AMD.

## 2. Material and Methods

### 2.1. Human Retinal Pigmented Epithelial Cell Line (ARPE-19)

ARPE-19 cells obtained from American Type Culture Collection (ATCC, Manassas, VA, USA), passages 12–19, were grown on gelatin-coated wells in Dulbecco’s modified Eagle’s medium–nutrient mixture F-12 (DMEM/F-12, Thermo Scientific, Wyman, MA, USA) supplemented with 10% Fetal bovine serum (FBS, Atlantic Biological, Norcross, GA, USA) and 1% penicillin/streptomycin (PS). ARPE-19 cells were then shifted to the serum free media 4–8 h before treatment with BMP2 (R&D Systems, cat# 355-BM/Carrier free (CF)) or BMP4 (R&D Systems, cat# 314-BP/CF) or vehicle (control) used to reconstitute BMP2/4 (4 mM HCl) in serum free media.

### 2.2. Measurement of ARPE-19 Cell Barrier Function

Effects of BMP2 or 4 on barrier function of ARPE-19 were evaluated by monitoring changes in Trans-Cellular Electrical Resistance (TER). Normalized TER was recorded by Electric Cell-substrate Impedance Sensing (ECIS^®^Zθ (theta)) instrument (Applied Biophysics Inc, Troy, NY, USA) as previously described [27,28,29]. Briefly, a 96-wells arrays (catalog # 96W20idf PET, Applied Biophysics Inc) were coated with 100 µM cysteine (50 µL) for 30 min then with 0.02% gelatin (50 µL) for another 30 min. Thereafter, ARPE-19 cells were seeded in DMEM/F12 full media with 10% FBS and 1% PS. After ARPE-19 reached the confluency, indicated by a capacitance below 20F, they were serum starved then treated with BMP2 or 4. Resistance for each well was normalized by dividing the measured resistance at each time point by the baseline resistance acquired before the addition of the treatment and plotted as a function of time.

### 2.3. Immunofluorescence of Zonula Occludens (ZO)-1

RPE cells were stained with zonula occludens (ZO)-1 antibody according to our previous procedure [30]. Briefly, cells were fixed by paraformaldehyde (2%, 10 min) followed by one-hour blockage in normal goat serum. Next, cells were incubated overnight at 4 °C with antibody against ZO-1 (Invitrogen, 1:100) followed by an incubation with Oregon green labeled secondary antibody (1:500, Invitrogen, Eugene, OR, USA). Finally, images were taken with confocal microscopy excited with 488-nm laser line (LSM 510; Carl Zeiss, Thornwood, NY, USA).

### 2.4. Measurement of ARPE-19 Cell Migration

The migration of ARPE-19 cells under the effect of BMPs was assessed by ECIS Model 1600R (Applied BioPhysics) as described previously [27]. Briefly, 8W1E arrays were used and coated as described above before seeding ARPE-19 cells in full media. Cells were left undisturbed until fully attached forming a confluent monolayer indicated by a capacitance below 1nF. Thereafter, cells were serum starved overnight, then treated with or without BMP2 or 4 before wound-induction. The wound was induced by delivering AC current to small circular electrode (located on the central part of each well), to kill cells located on the circular electrode to create a wounded area in ARPE-19 cell monolayer. The wounded area was gradually healed by the migration of the viable cells surrounding the small electrodes, and this migration was monitored in a real-time by recording the time required for ARPE-19 cells to reform the monolayer, indicated by the recovery to 1nF capacitance.

### 2.5. Measurement of MMP Activities

Total MMP and MMP-2 activities in ARPE-19 protein extracts were measured using a Fluorometric SensoLyte 520 Generic MMP Assay Kits (AnaSpec, Fremont, CA, USA) according to the manufacturer’s instructions. Generally, these kits use a 5-FAM/QXL™520 fluorescence resonance energy transfer (FRET) peptide linked to different MMP substrates where the fluorescence of 5-FAM is quenched by QXL™520. MMP activities were quantified by measuring fluorescence intensity of 5-FAM released upon the cleavage by different MMPs at excitation/emission wavelengths = 490 nm/520 nm.

### 2.6. Determination of Tissue and Circulating Levels of BMP2 and BMP4 in Retina, RPE, and Serum Samples from nAMD Donors

The retinas, RPE/choroid, and serum samples were taken from AMD patients (2 males and 3 females; age 83 ± 6.5) and Controls (6 males and 4 females; age 74 ± 9.6), all of Caucasian European ancestry. Comorbidities for this set of donors both disease and normal include hypertension and dyslipidemia and the cause of death was myocardial infarction. Donor tissues were collected, ascertained, processed and phenotyped as previously described for the Utah protocol [31]. Briefly, human eyes and blood samples were obtained from donors who have given consent for postmortem tissue donation and who reside within one hour’s car-ride away from the Utah Lions Eye Bank (at the John A. Moran Eye Center in Salt Lake City, UT, USA). This one-hour travel determination is an operational decision made to circumvent changes in gene expression and RNA quality. Donors with a history of herpes simplex virus (HSV) or human immunodeficiency virus (HIV) were excluded. For rigorous standardized postmortem ocular phenotyping, spectral-domain optical coherence (SD-OCT) tomography together with color fundus photography were used to image normal and diseased donor eyes as previously described [31] using the clinically derived modified Age-Related Eye Disease Study severity grading scale (AREDS 1, AREDS 2, AREDS3 (intermediate), AREDS 4a (geographic atrophy), AREDS 4b (nAMD)) [32]. Donor’s eyes with any history of diabetes, intermediate nonexudative AMD, or geographic atrophy were not used for this study. Thereafter, we followed our previously published standardized dissection protocol [31] to dissect donors’ eyes and to reliably isolate the RPE/choroid from the retina. To do so, we used an 8-mm disposable biopsy punch (Integra, USA) placed over the fovea followed by a 6-mm punch to cut a button of RPE/choroid from within the 8-mm punch. This smaller punch of 6 mm reduces retina contamination to the RPE/choroid [33] personal communication]. In the end, retinal tissues were isolated from the underlying RPE/choroid tissues under a dissecting microscope and both tissues were preserved. For a detailed protocol please see [31].

This protocol was approved by the Institutional Review Board (IRB) (IRB 00052879) at the University of Utah and conforms to the tenets of the Declaration of Helsinki. To determine the tissue levels of the BMP family, total RNAs from retina and RPE/choroid were then extracted and converted to cDNA to prepare sequencing libraries, which were read on Illumina Hiseq2500 and mapped back to the human genome hg19 (GRCh37) as previously described [34]. Transcript abundance was determined a previously described [34]. For measuring the circulating levels of BMP2 and BMP4, from human donor patients, ELISA Kits (Catalog #DBP200 and DBP400, respectively, R&D Systems, Minneapolis, MN, USA) were used per the manufacturer’s instructions. Briefly, Assay Diluents were applied first to each well. Then, equal amount of serum protein per samples (1 mg) have been added and incubated for 120 min. Next, wells were washed then a secondary antibody conjugated with horseradish peroxidase was added and the plate was incubated for another 120 min. The wash step was repeated again, substrate solution was added, and the wells were incubated for 30 min. At the end, stop solution was added and the optical density was measured by a microplate reader (Synergy HI Hybrid Reader, BioTek) at 450 nm against standards. The results were presented in pg/g serum protein.

### 2.7. Data Analysis

Statistical analyses were conducted using GraphPad Prism 8. Differences among experimental groups were evaluated by using the two-tailed *t* test or one-way analysis of variance (ANOVA) followed by Tukey test. Categorical variables and the difference in serum levels of BMP2/4 as evaluated by Chi Square test. A *p* value < 0.05 was considered statistically significant. For detection of differentially expressed genes (DEGs) within BMP family, raw read count was used as input for DEG analysis with the DEseq2 package. The fold-change of DEG was determined using the raw read counts normalized with total read counts using fragments per kilobase per million sequenced reads (FPKM) and defined as genes that had expression with false discovery rate (FDR) less than 0.05 after Benjamini–Hockberg correction.

## 3. Results

### 3.1. Effects of BMP2 and BMP4 on RPE Barrier Function

**Figure 1:**
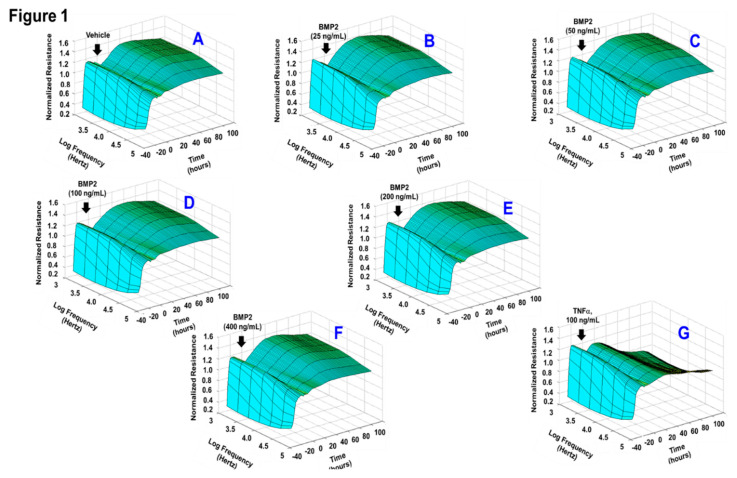
Three-D model representation for the effect of bone morphogenetic protein (BMP)2 on ARPE-19 barrier functionality. By using a real-time monitoring of transepithelial electric resistance (TER) as an indicator of barrier integrity, BMP2-treatment was initiated after ARPE-19 cells formed confluent mature monolayersindicated by the plateau in TER, *y*-axis shown in the representative 3D model. Thereafter, monolayers of ARPE-19 cells were treated with BMP2 at different concentrations (0, 25, 50, 100, 200, or 400 ng/mL; (**A**–**F**), respectively) and the barrier integrity was monitored over a 100-h period (represented on the *z*-axis) as well as across 9 frequencies (250, 500, 1000, 2000, 4000, 8000, 16,000, 32,000, and 64,000 Hz, represented as log values on the *x*-axis). As shown in (**A**–**F**), BMP2 did not affect the barrier integrity of ARPE-19 at any of tested concentration compared to the positive control cells, which were treated with TNFα at 100ng/mL (**G**); *n* = 4–6.

**Figure 2:**
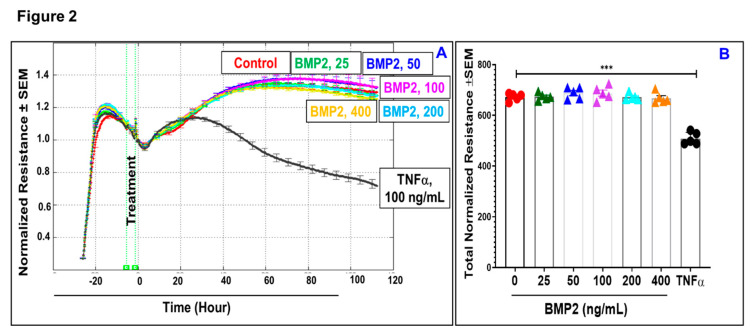
Normalized resistances of BMP2-treated ARPE-19 cells measured at a frequency of 4000 Hz by electrical cell-substrate impedance sensing (ECIS) (**A**), and the statistical analysis (**B**). Our experiments were carried out after ARPE-19 cells formed confluent mature monolayers indicated by a plateau of electronic resistance, where the resistance reached 1000 ohms. However, to monitor changes in the resistance in real time, the data are presented as normalized TER resistance calculated by dividing the resistance of each well measured in ohms at each time point by the baseline resistance (ohms) acquired before the addition of the BMP2 and plotted as a function of time. There was no significant drop in the resistances of ARPE-19 cells with direct BMP2-treatment in the range of (0–400 ng/mL) over the experimental period as compared to positive control of TNF-αtreated ARPE-19 cells. Red circles: control; green triangles: BMP2 (25 ng/mL); blue triangles: BMP2 (50 ng/mL); purple triangles: BMP2 (100 ng/mL); aqua triangles: BMP2 (200 ng/mL); orange triangles: BMP2 (400 ng/mL); black circles: TNFα (100ng/mL). Results are presented as mean ± SEM, *n* = 4–6; *** = *P* < 0.001.

**Figure 3:**
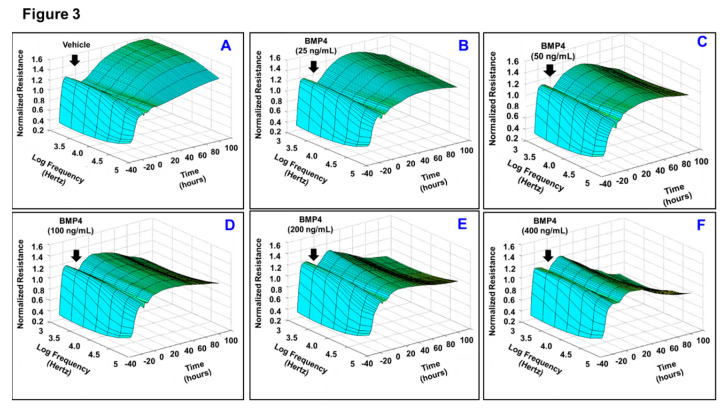
Three-D model representation for the effect of BMP4 on ARPE-19 barrier functionality. BMP4-treatment was initiated after ARPE-19 cells formed confluent monolayers indicated by the plateau in the resistance, *y*-axis shown in the representative 3D model. Thereafter, monolayers of ARPE-19 cells were treated with BMP4 at different concentrations (0, 25, 50, 100, 200, or 400 ng/mL; (**A**–**F**), respectively) and the barrier integrity was monitored over a 100-h period (represented on the *z*-axis) as well as across 9 frequencies (250, 500, 1000, 2000, 4000, 8000, 16,000, 32,000 and 64,000 Hz, represented as log values on the *x*-axis). As shown in (**A**–**F**), BMP4 reduced the resistance of ARPE-19 cells in a dose dependent manner compared to the vehicle-treated control cells over the experimental period and across all tested frequencies; *n* = 4–6.

**Figure 4:**
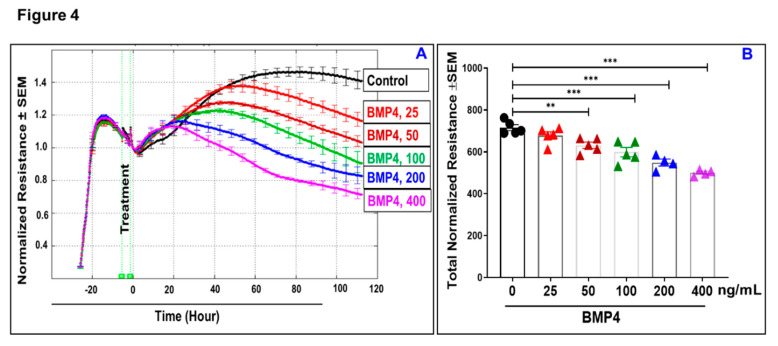

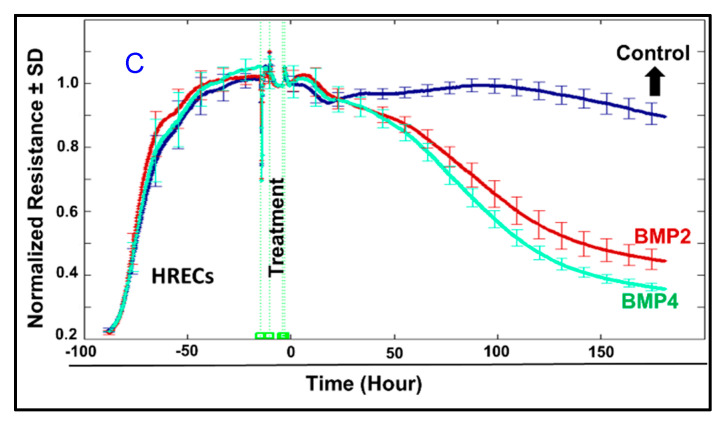
Normalized resistances of BMP4-treated ARPE-19 cells measured at a frequency of 4000 Hz by ECIS (**A**), and the statistical analysis (**B**). Our experiments were carried out after ARPE-19 cells formed confluent mature monolayers indicated by a plateau of electronic resistance, where the resistance reached 1000 ohms. However, to monitor changes in the resistance in real time, the data are presented as normalized TER resistance calculated by dividing the resistance of each well measured in ohms at each time point by the baseline resistance (ohms) acquired before the addition of the BMP4 and plotted as a function of time. A significant drop in the resistances of ARPE-19 cells with direct BMP4-treatment in the range of (50–400 ng/mL) over the experimental period as compared to vehicle-treated control ARPE-19 cells. Results are presented as mean ± SEM, *n* = 4–6/group; ** = *P* < 0.01; *** = *P* < 0.001. Black circles: control; red triangles: BMP4 (25 ng/mL); dark-red triangles: BMP4 (50 ng/mL); green triangles: BMP4 (100 ng/mL); blue triangles: BMP4 (200 ng/mL); purple triangles: BMP4 (400 ng/mL). (**C**) To ensure that the used BMP2 and 4 were active proteins, we confirmed their activity in other retinal cell-type system using human retinal endothelial cells (HRECs). As shown in the Figure, both BMP2 and 4 remarkably affected HREC’s barrier integrity. Results are presented as mean ± SEM, *n* = 6/group.

Given the fact that loss of monolayer integrity is closely related to the RPE dysfunction in nAMD, a functional assay was carried out in vitro to investigate whether BMP2 or BMP4 disrupts barrier function of ARPE-19 cells using a real-time monitoring of transepithelial electric resistance (TER) as an indicator of barrier integrity. The treatment was initiated after ARPE-19 cells formed confluent mature monolayers indicated by the plateau in TER, *y*-axis shown in the 3D model (Figure 1). Thereafter, monolayers of ARPE-19 cells were treated with BMP2 at different concentrations (0, 25, 50, 100, 200, or 400 ng/mL; Figure 1A–F, respectively) and the barrier integrity was monitored over a 100-h period (represented on the *z*-axis) as well as across 9 frequencies (250, 500, 1000, 2000, 4000, 8000, 16,000, 32,000, and 64,000 Hz, represented as log values on the *x*-axis). As shown in Figure 1A–F, BMP2 did not affect the barrier integrity of ARPE-19 at any of tested concentration compared to the positive control cells, which were treated with TNFα at 100 ng/mL (Figure 1G). These observations were further supported by the statistical analysis of all normalized resistances measured at a frequency of 4000 Hz, a commonly used frequency to assess barrier functionality [35] (Figure 2). As shown in Figure 2A,B, there was no significant drop in TER of ARPE-19 cells with direct BMP2-treatment in the range of (0–400 ng/mL) over the experimental period as compared to TNFα-treated RPE cells. On the contrary to BMP2, BMP4 reduced TER of ARPE-19 cells in a dose dependent manner compared to the vehicle-treated control cells over the experimental period and across all tested frequencies (Figure 3A–F). This is clearly depicted in Figure 4A,B, where resistance measurements taken at 4000 Hz showed a significant drop in TER of ARPE-19 by BMP4-treatmet in a dose dependent manner starting at 50 ng/mL. To ensure that the used BMP2 and 4 were active proteins, we confirmed their activity in other retinal cell-type system using human retinal endothelial cells (HRECs). As shown in Figure 4C, both BMP2 and 4 remarkably affected HREC’s barrier integrity. This is in contrast to their differential effect on the barrier integrity of ARPE-19.

**Figure 5:**
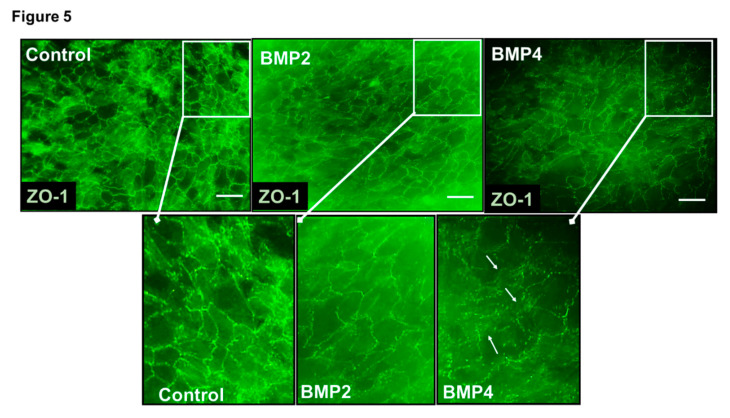
Representative photographs of ZO-1 immunofluorescence (green) in ARPE-19 cells treated with BMP2 or BMP4 versus vehicle-treated controls. Enlarged pictures were shown in squares and the arrows are pointing to the punctate pattern alteration in ZO-1 distribution at cellular border of BMP4-treated ARPE-19 cells; *n* = 4/group; scale bar = 100 µm.

The finding that BMP4 but not BMP2 significantly reduced TER of ARPE-19 has been further substantiated by testing the differential effects of BMP2 and BMP4 at 50 ng/mL on modulating the expression of ZO-1, a tight junction protein that is crucial in maintaining the barrier function. Concordantly, as shown in Figure 5, a smooth and continuous staining for ZO-1 along the intercellular borders of ARPE-19 cells was seen in vehicle-treated controls. However, treating ARPE-19 with BMP4 not BMP2 caused a punctate pattern alteration in ZO-1 distribution at cellular border, displaying the discontinuity of tight junctions. Taken together, these data demonstrate that BMP4 but not BMP2 is disrupting the barrier functionality of ARPE-19.

### 3.2. BMP4 But Not BMP-2 Increases ARPE-19 Migration Rate

**Figure 6:**
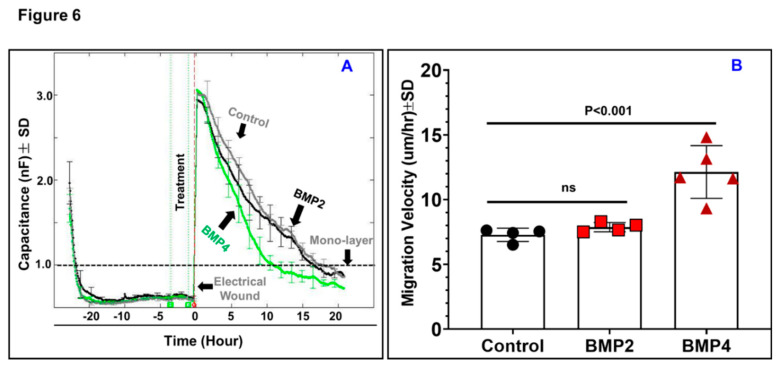
Migration assay of ARPE-19 cells using ECIS. (**A**) ARPE-19 cells treated with BMP2 and BMP4 (50 ng/mL each) versus control. After electrical wound creation at vertical dotted red line, migration of ARPE-19 cells was measured by their ability to re-form a monolayer indicated by capacitance of 1nF (Horizontal dashed line). (**B**) The migration velocity calculated by dividing the total distance that ARPE-19 cells migrated on the radius of the electrode which is 125-μm divided by the time required for recovering 100% of normalized capacitance, the recovery point. The cells treated with BMP4 re-formed a mono-layer significantly faster than BMP2 and the control groups. Meanwhile, migration of the BMP2 treated ARPE-19 cells showed no significant difference versus the control group. Black circles: control; Red squares: BMP2; Red Triangles: BMP4. Results are presented as mean ± SD; *n* = 4–5 per group; ns = non-significant.

Intraretinal RPE migration into the neurosensory retina has been reported to be an indicative of neovascular progression in AMD [22]. To this end, we tested the direct effects of BMP2 or BMP4 on ARPE-19 migration, a crucial step for neovascular formation, using in vitro ECIS assay, which replaces the traditional “scratch” assay. Our data showed that BMP4 but not BMP2 remarkably increased ARPE-19 migration rate. This is graphically depicted in Figure 6A, where the wounding phase was induced by an elevated field pulse (2.5 V at 40 kHz) for 20 s. As a result, ARPE-19 cells lift from the circular gold electrode and consequently the capacitance increased to 3nF, which is the capacitance associated with the cell-free electrode. The capacitance then dropped quickly during the healing phase due to RPE migration to reform the monolayer over the electrode. By ~10 h, this process was nearly completed under BMP4 treatment, while it took ~17 h for BMP2- or vehicle-treated RPE cells to reform the monolayer. The migration velocity was then calculated by dividing the distance that ARPE-19 cells travelled on the radius of the gold electrode, which is 125 μm, by the time required for recovering 1nF capacitance, the confluence point. The rate of migration of BMP4-treated ARPE-19 cells was significantly higher (*P* < 0.01) than the rate obtained by BMP2-treated cells or vehicle-treated control by ~2-fold (Figure 6B), indicating a differential effect of BMP2 and BMP4 on ARPE-19 functionality.

### 3.3. BMP4 But Not BMP2 Increases Matrix Metalloproteinase (MMP) Activity in ARPE-19

**Figure 7:**
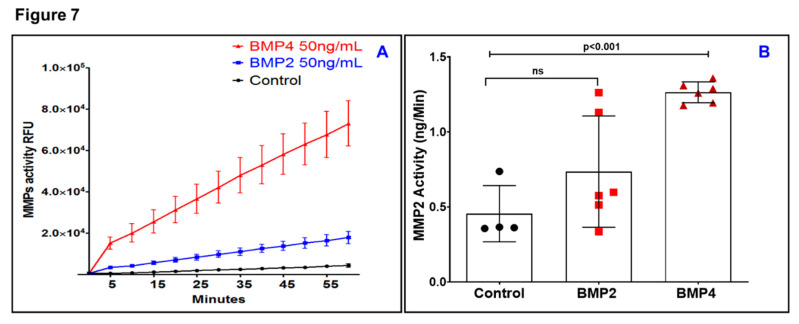
Sensolyte assay for kinetic analysis of matrix metalloproteinase (MMP) activity after BMP2- or BMP4-treatment. (**A**) A remarkable increase (~6 fold) in the slope of substrate cleavage by MMPs was seen against time for the BMP4-treated ARPE-19 cells in comparison with BMP2-treated ARPE-19 cells. (**B**) MMP2 activity was significantly (*P* < 0.001) increased after exposure to BMP4 but not BMP2; Black circles: control; Red squares: BMP2; Red Triangles: BMP4. Results are presented as mean ± SD; *n* = 4–6 per group.

After having shown that BMP2 and BMP4 have differential effects on ARPE-19 functionality, interest in understanding the underlying mechanisms has been expanded to study their effects on MMPs. Previous studies have pointed out that MMPs have a central role in mediating RPE cell migration by degrading extracellular matrix (ECM) to make a path for RPE to migrate [36,37]. Therefore, a causal relationship between the activation of MMPs and BMP2 or BMP4 was then investigated. To this end, the Sensolyte assay was used to conduct a kinetic analysis for total MMP activity after BMP2- or BMP4-treatment. As shown in Figure 7A, a remarkable increase (~6 fold) in the slope of substrate cleavage by MMPs was seen against time for the BMP4-treated ARPE-19 cells in comparison with BMP2-treated ARPE-19 cells. More specifically, MMP2 activity was significantly (*P* < 0.001) increased after exposure to BMP4 but not BMP2 (Figure 7B).

### 3.4. Tissue and Circulating Levels of BMP-Family in Donor Patients with nAMD

**Figure 8:**
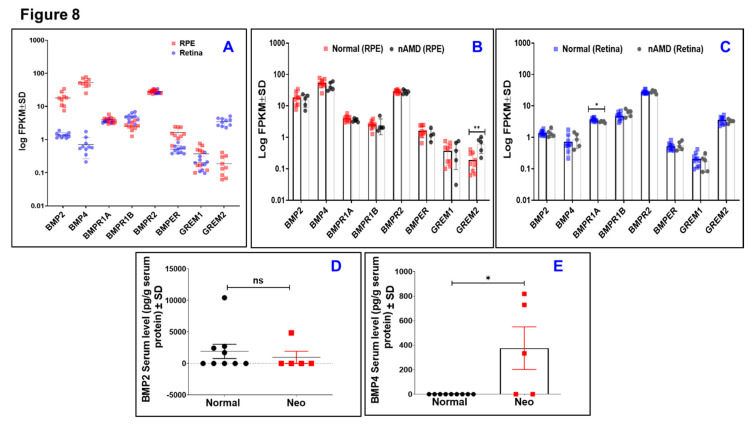
Tissue and circulating levels of BMP-family in donor patients with nAMD. (**A**) Local mRNA expression levels of BMP ligands (BMP2 and BMP4), three BMP specific receptors (BMPR1A, BMPR1B, and BMPR2), and three BMP regulators (BMPER, GREM1, and GREM2) in normal human retina versus RPE/choroid tissues determined by RNA-seq and represented as log of fragments per kilobase per million sequenced reads (FPKM); *n* = 9–10 per group. (**B**) Log FPKM expression of BMP-family in RPE/choroid from nAMD and normal groups; ** *=* adjusted *P* < 0.01; *n* = 5–9 per group. (**C**) Log FPKM expression of BMP-family expression in the retina from nAMD and normal groups; * = adjusted *P* < 0.05; *n* = 5–10 per group. (**D**,**E**) Circulating BMP2 and BMP4, respectively, in serum samples from nAMD donors versus normal donors determined by ELISA Assay. The difference in serum levels of BMP2 and BMP4 has been evaluated by Chi Square test. * represents Chi Square *P*-Value which was statistically significant (< 0.05). Results are presented as mean ± SD; *n* = 5–9 per group. AMD patients (2 males and 3 females; Age 83 ± 6.5) and Controls (6 males and 4 females; Age 74 ± 9.6).

To obtain a systematic view on BMP signaling in nAMD, we first determined the local mRNA expression levels of BMP ligands (BMP2 and BMP4), three BMP specific receptors (BMPR1A, BMPR1B, and BMPR2), and three BMP regulators (BMPER, GREM1, and GREM2) in normal retina versus RPE/choroid. As shown in Figure 8A, detectable expression levels of BMP regulators and receptors were seen in RPE/choroid at comparable levels to these observed in retina tissues amongst normal donor samples. After Benjamini–Hockberg correction, intriguingly, BMP2 and BMP4 are expressed in the macular RPE/choroid but not in the macular retina. This is further substantiated by the in vitro measurement of endogenous BMP4 level in ARPE-19, which reached 2.5 ± 0.22 ng/mL. This concentration is far less than the concentration range (50–400 ng/mL) required to induce barrier dysfunction. Next, we determined the changes in local BMP-family expression in RPE/choroid and retinas of nAMD versus normal samples. As shown in Figure 8B, the expression of BMP-family, including BMP2 and BMP4, in RPE did not notably differ between those with neovascular AMD and those as controls except for GREM2 which significantly increased in RPE/choroid of nAMD group (*P* < 0.01). Similarly, the expression of BMP-family in retina did not change between nAMD and normal controls except for BMPR1A which significantly decreased (15%, *P* < 0.05) in the retinas of nAMD group (Figure 8C). On the other hand, and in contrary to what has been observed for unchanged retinal and RPE/choroid levels of BMP2 and BMP4 in nAMD, the circulating BMP4 but not BMP2 was significantly (*P* < 0.05) increased in serum samples from nAMD donors (Figure 8D,E). Collectively, these data suggest that circulating BMP4 but not circulating BMP2 or local BMP4 is correlated with the development of nAMD.

## 4. Discussions

RPE dysfunction has been recognized as a potential cellular starting point contributing to AMD pathogene [20,38,39,40,41,42]. However, the detailed mechanism by which RPE dysfunction occurs in nAMD remains unclear. In the current study, we presented for the first time a new concept that BMP4 rather than BMP2 is a contributing factor to RPE dysfunction in nAMD. The following evidence supports this conclusion: (a) Circulating BMP4 but not BMP2 was significantly detected in serum sample from nAMD donors; (b) BMP4 but not BMP2 disrupted the barrier functionality of RPE in a dose dependent manner; (c) BMP4 but not BMP-2 increased RPE migration rate and enhanced the activity of matrix metalloproteinase activity in RPE.

Our decision to study BMP2 and BMP4 in nAMD was based upon our RNA-seq data from nAMD donors which showed BMP2 and BMP4 are the most abundant proteins among the BMP family expressed in RPE/choroid rather than in retina. BMP2 and BMP4 are initially synthetized as inactive precursors that are converted to mature active form by the proteolytic cleavage. These mature forms are regulated by endogenous antagonists such as BMPER, noggin, and gremlins [43,44]. Although BMP2 and BMP4 have important roles in the development of osseous tissues, their uncontrolled levels were associated with the progression of many diseases. For example, they have been identified as risk factors for type-2 diabetes, coronary artery diseases, diabetic retinopathy and endothelial cell dysfunction [43,45,46,47]. Nevertheless, few studies have investigated the role of BMP2 and BMP4 in tissues affected by AMD. Prior data from western blot analysis on RPE and choroid in the macular region dissected from two patients with early AMD demonstrated an increased expression of BMP4 [48]. This initial observation has been supported by additional histopathologic studies showing an increased BMP4 expression in retinal sections from donors with early AMD in the areas of RPE and the Bruch membrane directly adjacent to hard and soft drusen [48], whereas in control samples from patients without AMD the immunohistochemical expression of BMP4 was undetected in RPE. Additionally, in retinal sections from three donors with geographic atrophy, BMP4 was detected in areas adjacent to RPE loss and in close proximity to the choroidal vasculature [48]. Furthermore, in the neovascular AMD lesions that had progressed to a fibrous scar, the expression of BMP4 increased in the RPE [49]. In contrast, BMP4 expression was not seen in the RPE associated with choroidal neovascular membranes in nAMD [49] nor in aqueous humor samples of nAMD patients [50]. Consistent with these previous studies, we found that BMP4 mRNA is expressed in RPE/choroid isolated from normal donor eyes but its tissue expression level did not change in nAMD donor eyes However, our study is the first to show that the levels of circulating but not local BMP4 were increased in nAMD, suggesting that imbalance between systemic and local BMP4 has the potential to cause RPE dysfunction in nAMD. It is worthy to mention that although our study is limited by small sample size, the population are rigorously phenotyped [31,34,51] and no published repository to date has correlated systemic and local tissue levels of BMPs from the same donor as our current study did. However, further research using independent cohorts is required to validate the study findings.

Surprisingly, the BMP4 was below the detection limit of 3.68 pg/mL in the sera of the control group which might seem contrary to other reports that showed variable levels of BMP4 in the serum of normal human subjects and these levels are over a wide range between 0.6 pg/mL to 127 ng/mL Such differences can be explained by the usage of different ELISA kits with different cut off values. For example, Yurekli et al. [46] reported a very high level of BMP4 (127 ng/mL) in the sera of normal human subjects by using the local commercial ELISA assays (Shanghai, China). On the other hand, Son et al. [52] used an ELISA-based system similar to what we have used in this study and reported a very low level of BMP4 in the serum of normal subjects (0.63 ± 0.41 pg/mL).

Accordingly, the direct effects of BMPs on RPE functionality has been explored through in vitro studies using ARPE-19, an RPE cell line, and our data further underscore the potential role of BMP4 but not BMP2 in dysregulating ARPE-19 function and in turn the development of AMD. Of note, the correlation between in vitro and in vivo BMP4 concentrations is quite difficult since the plasma level is not the only contributing factor to the activity of circulating BMP4 but also the exposure time is another important factor and this is relatively hard to be accurately determined in a retrospective type of study such as the one involved here. However, by assuming the total amount of protein in the blood is constituting 6–8% of the plasma volume (which is around 3500 mL) then the total amount of BMP4 in circulation could be calculated from the detected level which is ~0.4 ng per 1g serum protein. By doing so, the total amount of BMP4 would be (0.4 × 3500 × (0.06 to 0.08)) = 84 to 112 ng which corresponds to a concentration of ∼250–400 ng/mL contained in individual wells of 96-well plate with 300 μL volume. Furthermore, this concentration range of BMPs was seen in our previous publication and showed consistent permeability and leukostasis effects in addition to the activation of BMP signaling [47]. Others have used a similar concentration to induce in-vitro and in-vivo permeability effects [53,54]. Therefore, a dose–response study was performed here to disentangle the effect of BMP4 on the barrier integrity of RPE over the concentration range covering most experimental and clinical cases (25 and 400 ng/mL).

We then evaluated signaling pathways mediated the BMP4-induced ARPE-19 permeability and migration by investigating its relationship with MMPs. Tissue remodeling in normal status requires MMP activity, which also shows positive correlation with AMD severity [55,56]. Previous studies showed contradictory results regarding the levels of MMPs in AMD. Chau et al., demonstrated increased levels of plasma MMP-9 in nAMD patients compared to healthy subjects [57], while other studies showed similar levels of MMP2 and MMP9 in both patient and control groups [58]. On the other hand, Hussain et al., showed a significant reduction in retinal levels of MMP9 in human donor eyes with AMD [55]. Interestingly, our current study demonstrates a significant increase in total MMP, specifically MMP2, activity in ARPE-19 cells subjected to BMP4 compared to BMP2-treatment or control groups. Contrary to this, pretreating RPE cells with BMP4 caused a significant decrease in MMP9 secretion [59]. This contradiction regarding the regulation of MMPs by BMP4 could be explained by the usage of different experimental models. For example, Xu et al. [59] tested the effect of BMP4 on MMP9 secretion in primary RPE cells isolated from fetal eyes after activation with TNF-α and linked this to the ability of BMP4 to inhibit choroidal neovascularization (CNV) in the experimental mouse model of laser-induced CNV using transgenic mice over-expressing BMP4. Since MMP-9 is able to act upstream of VEGF in human RPE cells [60], additional studies had shown that BMP4 has no effect on VEGF expression in primary RPE [61], while others reported that it increased VEGF secretion in ARPE-19 cells [26].

In conclusion, our results expand the current knowledge about the effects of BMP4 in inducing ARPE-19 barrier dysfunction and enhancing the migration of ARPE-19 cells and suggest that BMP4 at the systemic level but not BMP2 may represent a potential therapeutic target in nAMD. However, the relevance of these findings in other independent RPE sources, such as iPSC RPE or primary RPE, needs to be further investigated.
